# An improved biologically transparent illumination system that increases the accuracy of detecting the correct position of a nasogastric tube in the stomach

**DOI:** 10.1371/journal.pone.0295415

**Published:** 2023-12-07

**Authors:** Hanayo Masaki, Hirofumi Hirano, Junji Takahashi, Teppei Kamada, Eiji Masaki

**Affiliations:** Department of Anesthesiology, International University of Health and Welfare Hospital, Tochigi, Japan; Menzies School of Health Research, AUSTRALIA

## Abstract

The aim of this study was to determine whether an improved biologically transparent illumination system results in more reliable detection of the correct position of the nasogastric tube in surgical patients. In total, 102 patients undergoing general surgery were included in this prospective observational study. After general anesthesia, all patients were inserted a nasogastric tube equipped with an improved biologically transparent illumination catheter. Identification of biologically transparent light in the epigastric area indicated successful insertion of the nasogastric tube into the stomach. The position of the tube was confirmed by X-ray examination, and its findings were compared with those of the biologically transparent illumination system. We observed biologically transparent light in epigastric area in 87 of the 102 patients. X-ray examination revealed that the nasogastric tube was placed in the stomach in all of these 87 patients. Light was not observed in the remaining 15 patients; the tube position was confirmed in the stomach in 11 of these patients but not in the other 4 by X-ray examination. Illumination had a sensitivity of 88.8% and a specificity of 100%. Our results suggest that this improved biologically transparent illumination system increased the accuracy of detecting the correct position of a nasogastric tube in the stomach. X-ray examination is required to check the position of the nasogastric tube in patients when biologically transparent illumination light is negative.

## Introduction

A nasogastric tube (NGT) is often inserted in a variety of acute and chronic medical settings but is not free of complications. Misplacement of an NGT in the respiratory tract or esophagus can result in severe morbidity and mortality [1,2]. These complications are referred to as “never events” [3]. New approaches for identifying the correct position of an NGT have been developed to prevent such events, including a fiberoptic pH test device [4] and direct vision-guided [5] or electromagnetic trace-guided [6] tube placement. However, each approach comes with its own difficulties, including expense and need for expert training, which need to be resolved before routine clinical use. Therefore, X-ray examination is considered the gold standard for confirming the correct position of an NGT [7]; however, this method also has some disadvantages, including radiation exposure, a risk of misinterpretation, and being inconvenient for use in settings such as nursing homes [8].

Biologically transparent (BT) light is a safe, rapid, and simple technique for detecting the correct position of an NGT [9]. BT light emitted from the tip of a special type of catheter (i.e., a BT catheter) in the gastrointestinal tract is a highly bio-permeable red color and can be visualized from outside the body [10]. We have previously proposed that this technique is useful for identifying the correct position of an NGT in the stomach [9]. Recently, an improved BT light system was introduced to increase the accuracy of detection of the correct tube position in the stomach.

The purpose of this study was to determine whether the improved BT light system could achieve more favorable results than those obtained using the existing system. Therefore, an NGT equipped with the improved BT catheter was inserted in surgical patients under general anesthesia and the BT light was checked in the epigastric area.

## Materials and methods

The study had a prospective observational design and was conducted at a local university hospital with 400 beds. The study was approved by the ethics committee of the International University of Health and Welfare Hospital (No. 22-B-2) and conducted in accordance with the Ethical Guidelines for Clinical Studies in Japan, published by the Ministry of Health, Labour and Welfare, and the principles expressed in the Declaration of Helsinki. Written informed consent was obtained from all patients. The patients for this study were recruited from May 1^st^ 2023 to August 31^st^ 2023. All data underlying the findings presented in this report will be made available without restriction from the corresponding author upon reasonable request.

### BT light and catheter

**Fig 1:**
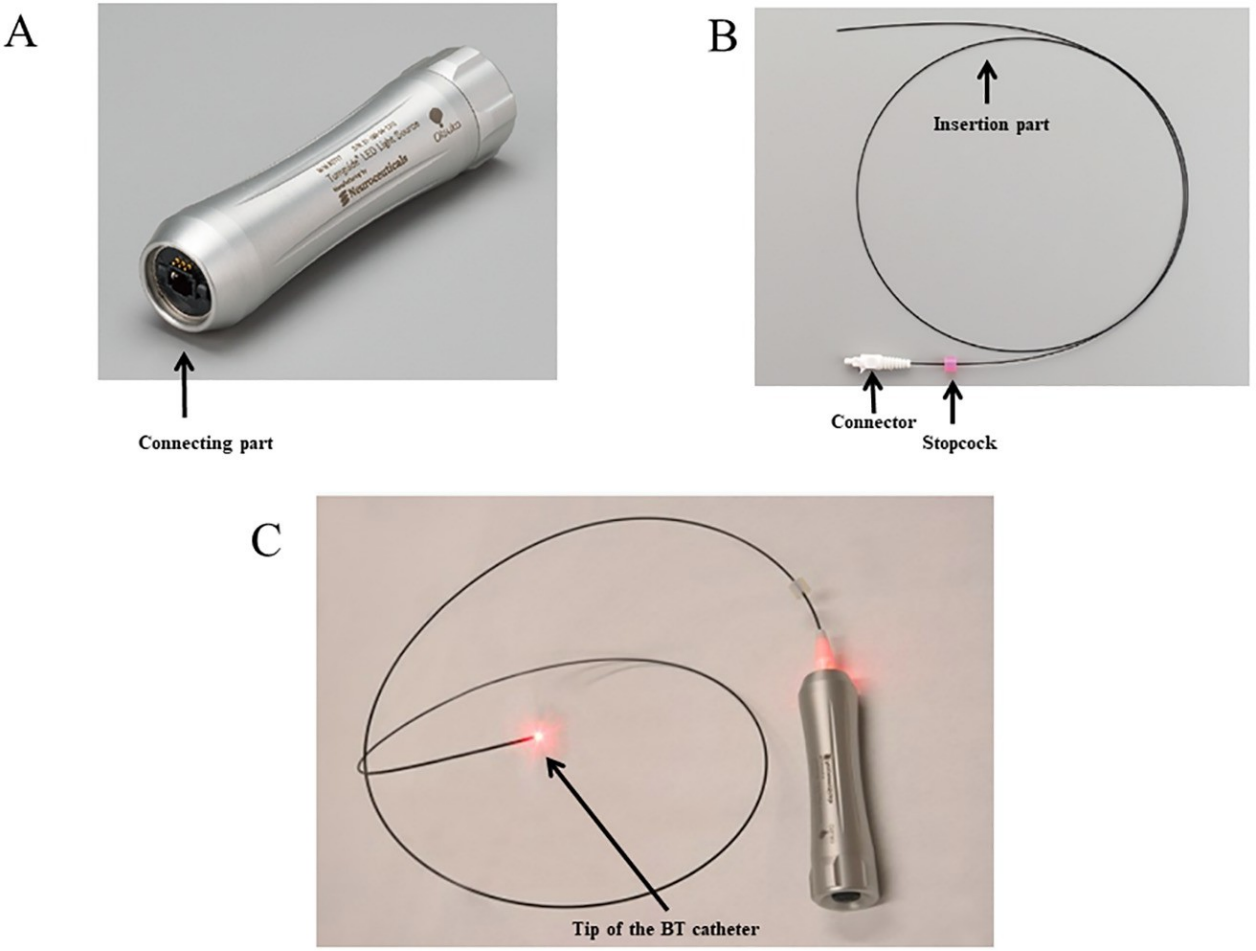
Components of the improved BT illumination system. (A) The improved biologically transparent (BT) light source. (B) The improved BT catheter. (C) BT light is transferred to the tip of the BT catheter. Reprinted from https://www.otsukakj.jp/med_nutrition/dikj/menu2/hoso/002864.php under a CC BY license, with permission from Otsuka Pharmaceutical Factory, Inc., original copyright 2021 for A and C, 2023 for B.

We investigated whether the novel BT light source and catheter could improve our ability to determine the correct position of an NGT in the stomach. The improved BT light source produces light that is 16% more intense, and the improved BT catheter has a beam angle that is 8% wider in comparison with the previously used light source and catheter. The properties of BT light and catheter have been described in detail elsewhere [9]. Briefly, the BT light source (Tumguide^®^ LED Light Source 13B1X10190000016; Neuroceuticals Inc., Tokyo, Japan, distributed by Otsuka Pharmaceutical Factory, Inc. Tokushima, Japan; Fig 1A) generates highly bio-permeable red light (wavelength, 660 nm), which is supplied to the BT catheter (Fig 1B). The BT catheter (Tumguide^®^ Fiber 13B1X10190000015; Neuroceuticals Inc., Tokyo, Japan, distributed by Otsuka Pharmaceutical Factory, Inc. Tokushima, Japan) has a diameter of 1.0 mm or 1.5 mm and transfers BT light to the tip of the BT catheter (Fig 1C). The position of the NGT tip can be identified by introducing the BT catheter into the NGT. BT light transferred to the tip of the BT catheter in the NGT in the stomach can be visually confirmed in the epigastric area from outside the body.

### Patients and procedures

This prospective observational study included 102 patients aged 18 years or older who required gastric decompression because of bag mask ventilation after induction of general anesthesia for general surgery. The anesthesiologist assessed whether decompression was achieved by insertion of an NGT (Salem Sump™/225AABZX00046000; Cardinal Health, Shizuioka, Japan). One of three sizes of NGT (14 Fr, 16 Fr, or 18 Fr) was chosen, and the first 5–10 cm of the tip was lubricated. The NGT equipped with the BT catheter was gently advanced from the nostril to a length determined by the nose-ear-xiphoid method. Care was taken not to apply too much force to the NGT during insertion. If the nasal passage was difficult to navigate, an oral approach was used. BT light was identified by the anesthesiologist with or without finger pressure. The pressure was applied with the index finger at 3–4 cm below the tenth rib on the midclavicular line in the epigastric area. After removal of the BT catheter from the NGT, the position of the NGT was confirmed by X-ray examination. Both the anesthesiologist and the surgeon interpreted the X-ray image, and the sensitivity and specificity of BT light were calculated using the X-ray examination results as the reference standard.

### Statistical analysis

All variables are expressed as the mean ± standard deviation. Sensitivity, specificity, positive predictive value, and negative predictive value were calculated to evaluate the diagnostic effectiveness of BT light.

Considering NGT would always be placed in the stomach when the BT light is detected in the epigastric area from outside the body, we hypothesized a positive predictive value of 100%. The required sample size was calculated by the Clopper-Pearson method [11] on the basis that the lower limit of the 95% confidence interval exceeds 95% of the positive predictive value [12] using SAS software (version 9.4; SAS Institute Inc., Cary, NC). The sample size required for the lower limit of the 95% confidence interval to exceed 95% is 72. The previous study [9] required 102 subjects to collect 72 samples. Therefore, we also collected 102 subjects in this study.

## Results

The characteristics of the 102 participants are shown in Table 1. Of the 102 patients, 55 were male and 47 were female. The mean age was 66.2 ± 13.9 years.

**Table 1:** Patient characteristics (N = 102).

Height (cm)	161 ± 10
Weight (kg)	60 ± 13
Age (years)	66 ± 14
BMI (kg/m^2^)	23 ± 4
Obese patients (%)	9

Data are expressed as the mean ± standard deviation.

**Fig 2:**
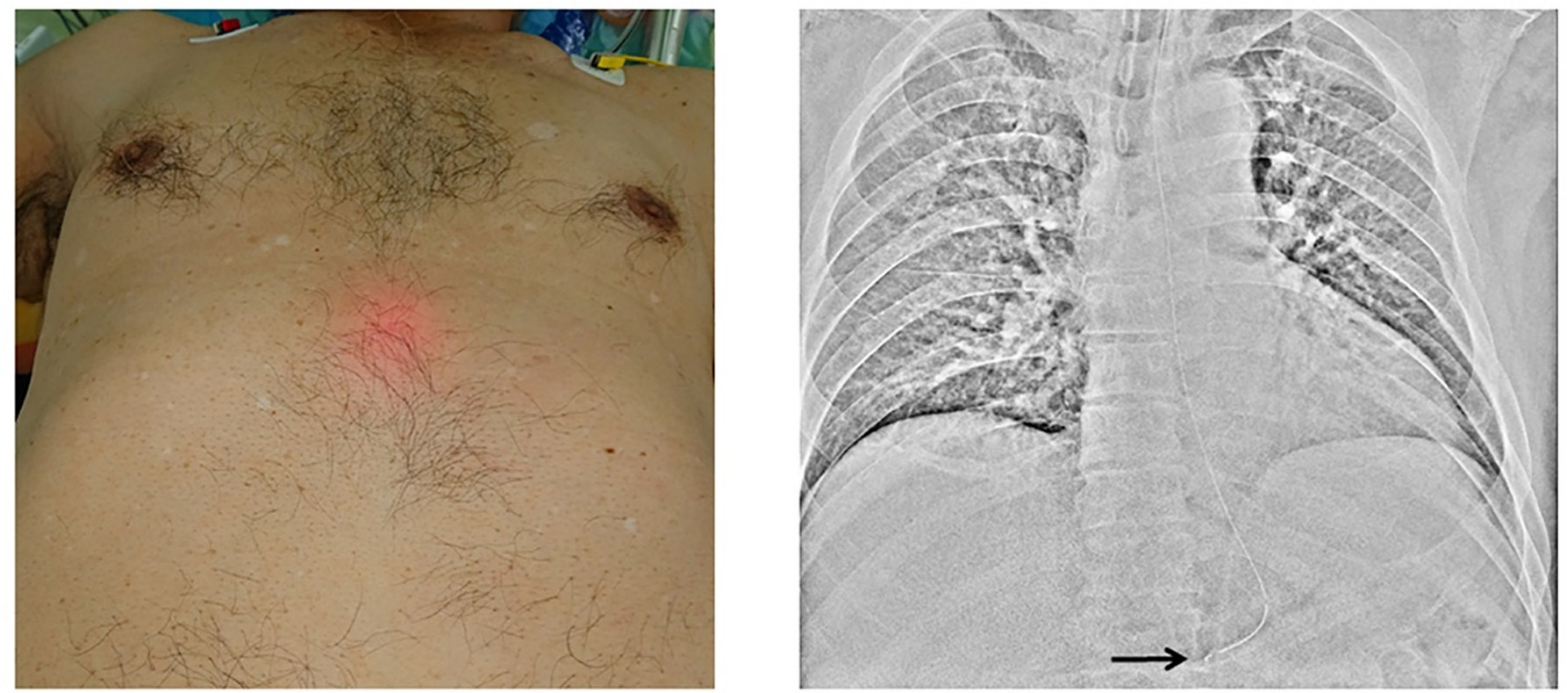
Positive biologically transparent light sign in the central epigastric area and corresponding X-ray image. The arrow indicates the tip of the nasogastric tube.

**Fig 3:**
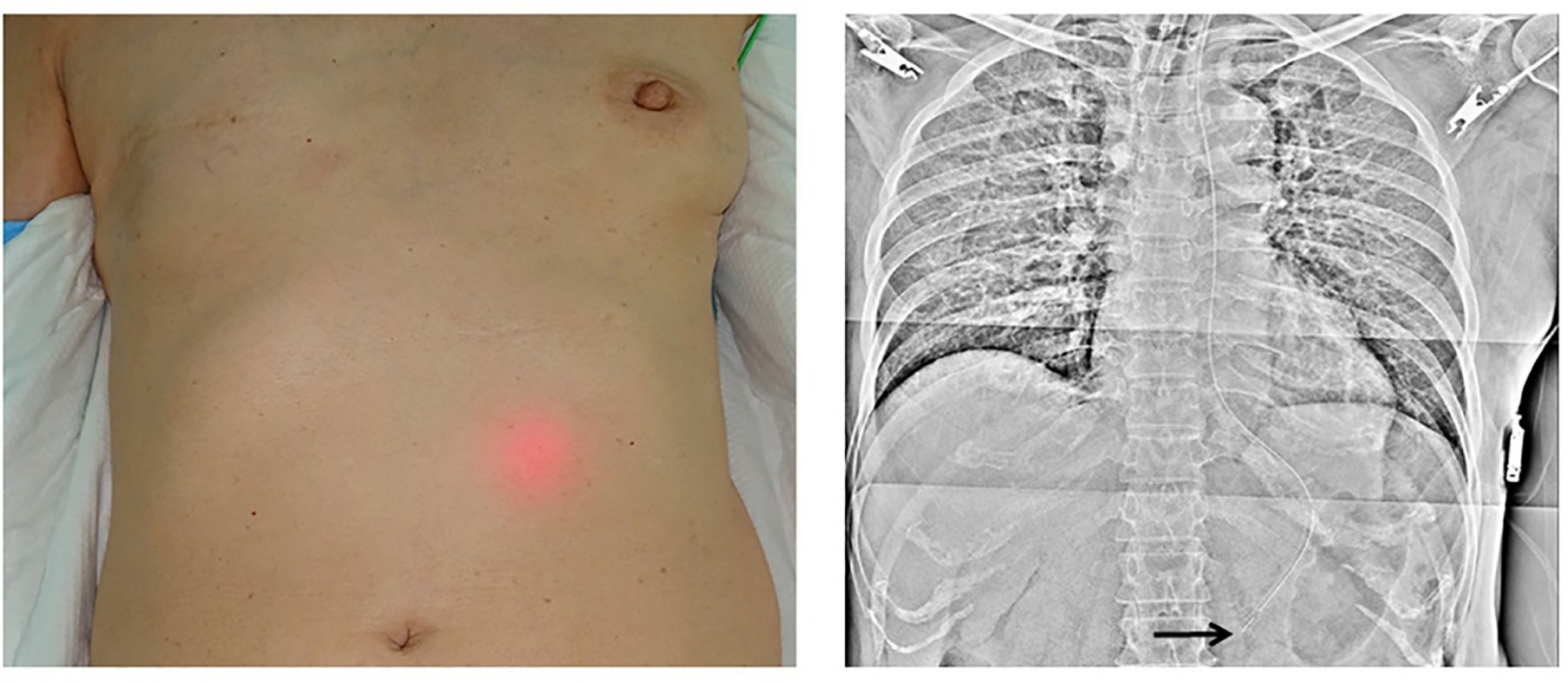
Positive biologically transparent light sign in the mid-epigastric area and corresponding X-ray image. The arrow indicates the tip of the nasogastric tube.

**Fig 4:**
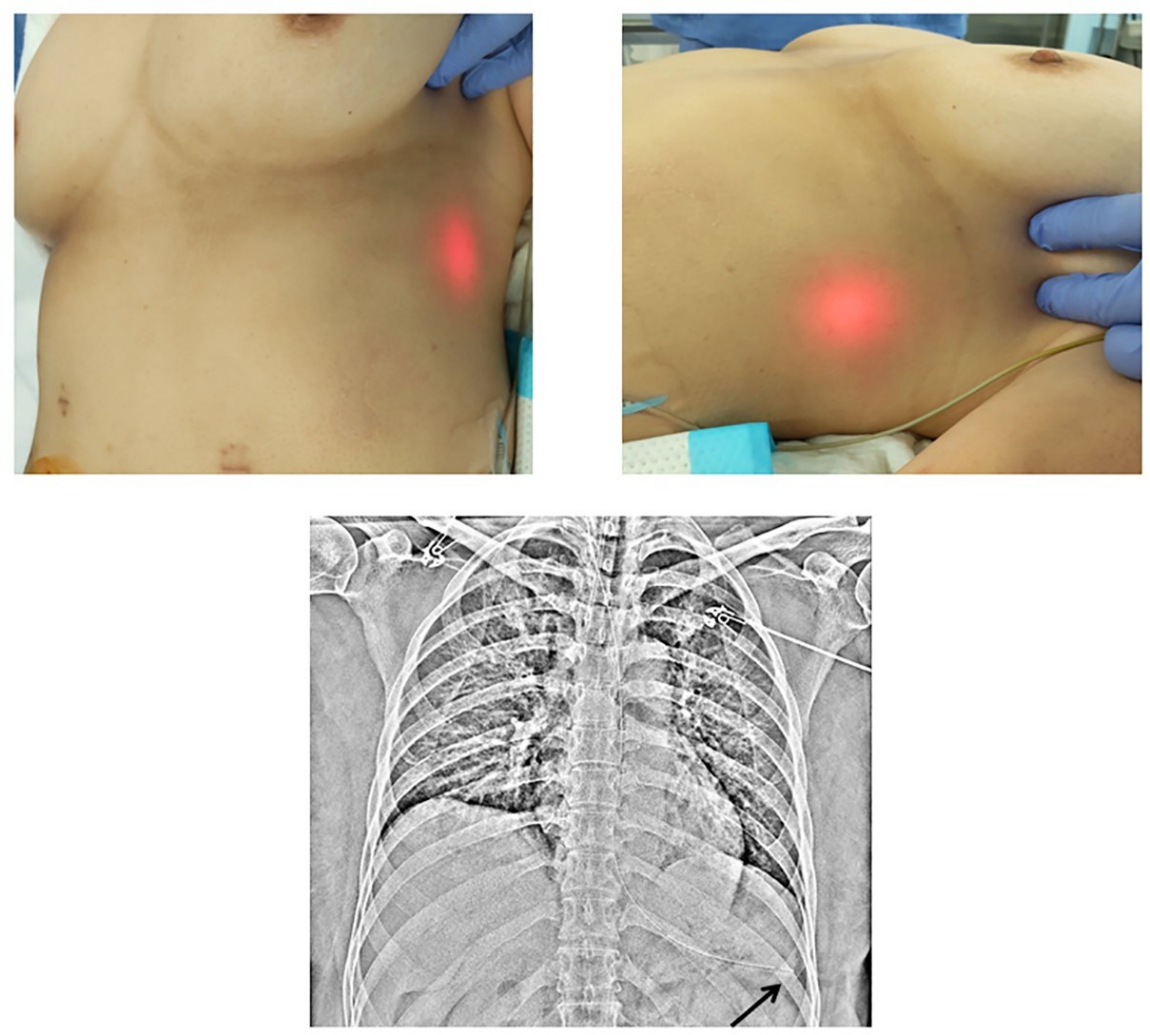
Positive Biologically transparent light sign in the lateral epigastric area and corresponding X-ray image. The arrow indicates the tip of the nasogastric tube.

We observed BT light in the epigastric area in 87 patients (Figs 2–4) but not in the remaining 15 patients. X-ray examination confirmed that the NGT was correctly placed in the stomach in 98 patients but was positioned at a site other than the stomach in 4 patients. X-ray examination also revealed that the NGT was definitely positioned in the stomach in all 87 BT light-positive patients. The NGT was confirmed to be present in the stomach in 11 of the 15 BT light-negative patients; however, in the remaining 4 patients, the NGT was located to be in the trachea (n = 1) or in the esophagus or esophagogastric junction (n = 3) (Table 2). The BT light test had a sensitivity of 88.8% and a specificity of 100%.

**Table 2:** Results of diagnostic tests.

Evaluation test	Reference test (X-ray)		Sensitivity (%)	Specificity (%)	PPV (%)	NPV (%)
	Correct position (n = 98)	Incorrect position (n = 4)				
**BT light**						
**Positive (+)**	87	0	89	100	100	27
**Negative (−)**	11	4				

BT, biologically transparent; NPV, negative predictive value; PPV, positive predictive value.

In obese patients (body mass index >27.5), the sensitivity of the BT light test was 77.8% (Table 3), which was lower than that for the patient group as a whole.

**Table 3:** Results of diagnostic tests in obese patients.

Evaluation test	Reference test (X-ray)		Sensitivity (%)	Specificity (%)	PPV (%)	NPV (%)
	Correct position (n = 9)	Incorrect position (n = 0)				
**BT light**						
**Positive (+)**	7	0	78	**	100	0
**Negative (−)**	2	0				

BT, biologically transparent; NPV, negative predictive value; PPV, positive predictive value. **Not calculable.

## Discussion

This study had two main findings. First, when the NGT was in the stomach, BT light was detected in 89% of patients; sensitivity was higher than that in the previous study (88.8% vs 77.4%) [9]. Second, when the NGT was not in the stomach, BT light was not identified in all patients; specificity was 100% and comparable with that previously reported [9]. These findings suggest that the improved BT light system would be more favorable for confirmation that an NGT is correctly positioned in the stomach and probably safe, potentially useful to avoid further x-rays, despite a small false-negative rate. Thus far, X-ray examination has been required to check the position of the NGT in BT light-negative patients.

The sensitivity of the improved BT light system was clearly higher in this study than in the earlier study [9], meaning that fewer of our patients in whom the NGT was correctly placed in the stomach were BT light-negative. The strong light emitted from the tip of the BT catheter needs to reach the abdominal wall for the BT light in the epigastric area to be observed from outside the body. In the past, we have speculated that one of the reasons for false-negative cases could be that the light intensity is too weak to be detectable from outside the body. The 16% more intense light and the 8% wider beam angle with the improved BT light system could provide more intense light from inside the stomach through the abdominal wall, resulting in increased detection of BT light when the NGT is in the stomach.

Nevertheless, false-negative cases still occurred in this study despite the 16% more intense light and the 8% wider beam angle with the improved BT light system. The intensity of BT light is most powerful in the direction perpendicular to the cross-section of the tip of the BT catheter [9]. If the cross-section of the BT catheter tip is heading in the dorsal and/or right direction and not in the ventral and/or left direction in the stomach, the intense BT light is pointed away from the abdominal wall. We found that BT light was not detectable from outside the body because the light intensity was inadequate as a result of the direction of the BT catheter. Indeed, in our preliminary study using fluoroscopic imaging, detection of BT light depended on the direction of the BT catheter (S1 Video). Development of a fully articulating catheter may prevent direction-related false-negative cases.

This study included 9 obese patients (body mass index >27.5). Sensitivity was lower in these patients than in the patient group as a whole (77.8% vs. 88.8%), although the statistical significance of this difference could not be examined because of the small number of obese patients. Given that the distance to which BT light can cross thick tissues has not been examined, the influence of obesity on BT light is unknown. In obese patients, the distance from the tip of the BT catheter to the body surface is greater than that in non-obese patients; therefore, the BT light would be too weak to reach the body surface in the epigastric area. Further studies are necessary to confirm whether the results of the present study can be applied to obese patients.

There were no false-positive cases in the present study or in the previous study. Namely, BT light could not be observed in the epigastric area when the NGT was not in the stomach. In addition, harmful events such as bleeding, regurgitation, and injury, related to insertion of the BT catheter were not observed in the present study. This finding suggests that the improved BT light system is safe. However, there were only 4 cases in which the NGT was not placed in the stomach in the present study and 9 in the previous study [9]. These small numbers of cases are insufficient to allow firm conclusions regarding safety. The NGT misplacement rate has been reported to be 1.6% in the lung and 5% in the esophagus [5]. Therefore, it would be difficult to accrue a large enough number of cases in which the NGT is incorrectly placed in the lung and esophagus to confirm the safety of the BT system. Studies in which an NGT equipped with a BT catheter is intentionally inserted into the lung or esophagus may be necessary to overcome the problem of the small number of cases of misplacement.

One of the advantages of the BT light system is that it is simple and convenient to use. This system does not need the extensive machinery and additional medical staff required when the position of the NGT is confirmed radiographically. The BT light system can be used in any medical setting where it is possible to use a BT catheter and its light source. Furthermore, unlike for ultrasonographic methods [13] or direct vision-guided tube placement [14], no special training and/or knowledge is required for the BT technique. These features of the BT light system suggest that this method could be useful in the community in settings such as nursing homes where X-ray and ultrasonography facilities are not readily available. All that is required is insertion of the BT catheter into the NGT, advancement of the NGT with the BT catheter, and watching for a red light in the epigastric area.

The limitation of this study is that only one type of NGT was inserted under general anesthesia and in the supine position. As mentioned above, different types of NGTs made from various materials are used for a variety of purposes in the clinical setting. Therefore, our present findings might not be reproducible when a different type of NGT is used without general anesthesia in a non-supine position because biological transparency could be affected under such conditions.

In conclusion, BT light was detected in 89% of patients when the NGT was in the stomach, and the NGT was always positioned in the stomach when BT light was identified in the epigastric area. These findings suggest that the improved BT illumination system described here increases the accuracy of detection of the correct position of an NGT in the stomach. X-ray examination is required to check the position of the NGT in BT light-negative patients.

## Supporting information

S1 Video Preliminary fluoroscopic study. (MP4)

S1 Table All relevant data. (XLSX)
